# Genetic diversity and population structure of the Sapsaree, a native Korean dog breed

**DOI:** 10.1186/s12863-019-0757-5

**Published:** 2019-08-05

**Authors:** Chandima Gajaweera, Ji Min Kang, Doo Ho Lee, Soo Hyun Lee, Yeong Kuk Kim, Hasini I. Wijayananda, Jong Joo Kim, Ji Hong Ha, Bong Hwan Choi, Seung Hwan Lee

**Affiliations:** 1 Division of Animal & Dairy Science, Chungnam National University, Daejeon, 34134 Republic of Korea; 2 Animal Genomics & Bioinformatics Division, National Institute of Animal Science, RDA, Wanju, 55365 Republic of Korea; 3 School of Biotechnology, Yeungnam University, Gyeongsan, 712-749 Republic of Korea; 4 School of Life Science, Kyungpook National University, Daegu, 41940 Republic of Korea; 5 Department of Animal Science, Faculty of Agriculture, University of Ruhuna, Matara, Sri Lanka

**Keywords:** Sapsaree, Genetic diversity, Population structure

## Abstract

**Background:**

The Sapsaree is a breed of dog (*Canis familiaris*) native to Korea, which became perilously close to extinction in the mid-1980s. However, with systematic genetic conservation and restoration efforts, this breed was rescued from extinction and population sizes have been gradually increasing over the past few decades. The aim of this study was to ascertain novel information about the genetic diversity, population structure, and demographic history of the Sapsaree breed using genome-wide single nucleotide polymorphism data. We characterized the genetic profile of the Sapsaree breed by comparison with seven foreign dog breeds with similar morphologies to estimate genetic differentiation within and among these breeds.

**Results:**

The results suggest that Sapsarees have higher genetic variance compared with the other breeds analyzed. The majority of the Sapsarees in this study share a discrete genetic pattern, although some individuals were slightly different, possibly as a consequence of the recent restoration process. Concordant results from analyses of linkage disequilibrium, effective population size, genetic diversity, and population structural analyses illustrate a relationship among the Sapsaree and the Tibetan breeds Tibetan terrier and Lhasa Apso, and a small genetic introgression from European breeds. The effective population size of the Sapsaree has contracted dramatically over the past generations, and is currently insufficient to maintain long-term viability of the breed’s genetic diversity.

**Conclusions:**

This study provides novel insights regarding the genetic diversity and population structure of the native Korean dog breed Sapsaree. Our results suggest the importance of a strategic and systematic approach to ensure the genetic diversity and the authenticity of the Sapsaree breed.

**Electronic supplementary material:**

The online version of this article (10.1186/s12863-019-0757-5) contains supplementary material, which is available to authorized users.

## Background

The domestic dog (*Canis familiaris*) is the most phenotypically diverse mammalian species, and one of the first animals to be domesticated by humans [1–3]. While dogs are the closest animal companion of humans, they are still used for specialized tasks including herding, hunting, retrieving, pulling sleds, and even for military tasks [4–6]. The gray wolf (*Canis lupus*) is the common ancestor of domesticated dogs, which have since been differentiated through artificial selection of the hugely diverse features of modern breeds [7, 8]. It has been hypothesized that the domestication of dogs began nearly 33,000 years ago in South East Asia. Ancestral canines accompanied humans in a migration to Africa and the Middle East around 15,000 years ago, and then to Europe around 10,000 years ago [6, 9–11].

Although evidence suggests dogs have been present on the Korean peninsula for a long period of time, the specifics of canine domestication are not well understood. Some have hypothesized that current dog breeds on the Korean peninsula were gradually introduced with the influx of humans. Today, there are more than 150 dog breeds on the Korean peninsula, and over 400 recognized dog breeds worldwide [12, 13]. Among the native Korean dog breeds, the Jindo, Sapsaree, and Donggyeong are protected as a designated ‘natural monument’ by the Korean government (Cultural Heritage Administration of Korea, #54, 368, and 540 respectively) [12, 14, 15]. The Poongsan breed was also designated as a natural monument during the Japanese colonial period (number 128), but the designation was removed by the Korean government in 1962 [12, 16].

The Sapsaree is a shaggy-haired and droopy-eared dog breed believed to reflect the character of the Korean people. They have a medium body size (54–62 cm in height) and two distinguishable coat colors: the ‘Chung’, or blue Sapsaree, and the ‘Hwang’, or yellow Sapsaree [12, 16, 17].

Historical evidence suggests that Sapsarees were used as military dogs by nobles of the Silla dynasty. Following the collapse of the unified Silla, Sapsarees were featured in the classical literary works of the Joseon dynasty and have since gained popularity throughout the Korean peninsula. Their disposition is friendly and gentle, and their loyalty has long been recognized [16, 18, 19].

The population size of Sapsaree was substantially decreased and became perilously close to extinction during the Japanese colonial period (1910–1945) and the Korean War (1950–1953). In 1969, a Sapsaree revival was initiated by Kyungpook National University, however the restoration process and systematic genetic conservation begin by 1985 at the Sapsaree Breeding Research Institute in Gyeongsan, South Korea. In 1992, the Sapsaree was registered as a national treasure of Korea and their breeding and sale were strictly regulated to protect the purity of the breed [17–23]. Current total Sapsaree population is approximately 4000 including the 500 dogs maintained at the Sapsaree Breeding Research Institute [19]. The existing Sapsaree population size is relatively small, and it will therefore be necessary to expand the population size to maintain the sustainability of the breed.

Understanding the genetic diversity of domesticated species is important to establish effective conservation decisions and management strategies [24, 25]. Advances in genome technology and the availability of high density genome-wide single nucleotide polymorphism (SNP) data have facilitated the characterization of genetic diversity and breed composition [26, 27]. Linkage disequilibrium (LD), effective population size (N_e_), and heterozygosity are parameters widely used to understand the genetic diversity of populations [24]. The evolutionary history of a population is estimated through LD, by estimating the non-random association between two genetic markers that results from various evolutionary and demographic processes [28, 29]. Another important parameter for estimating the demographic history of a population is N_e_, which estimates the rate of genetic drift, inbreeding, and the effects of evolutionary forces such as mutation, selection, and migration [30, 31]. Heterozygosity is also a widely used parameter to measure genetic variation within a population [23, 32]. Information regarding genetic diversity, LD, N_e_, and heterozygosity would therefore be useful for establishing a breeding program that avoids inbreeding while maintaining the breed purity of Sapsarees. However, there are a limited number of scientific studies on the genetic diversity of Sapsaree populations [20, 21, 23, 33]. In this study, we used high-density SNP data to estimate the genetic diversity of the Sapsaree. We characterized the genetic profile of the Sapsaree by comparison with seven foreign dog breeds with similar morphology and estimated the genetic differentiation within and among these breeds.

## Results

**Fig. 1:**
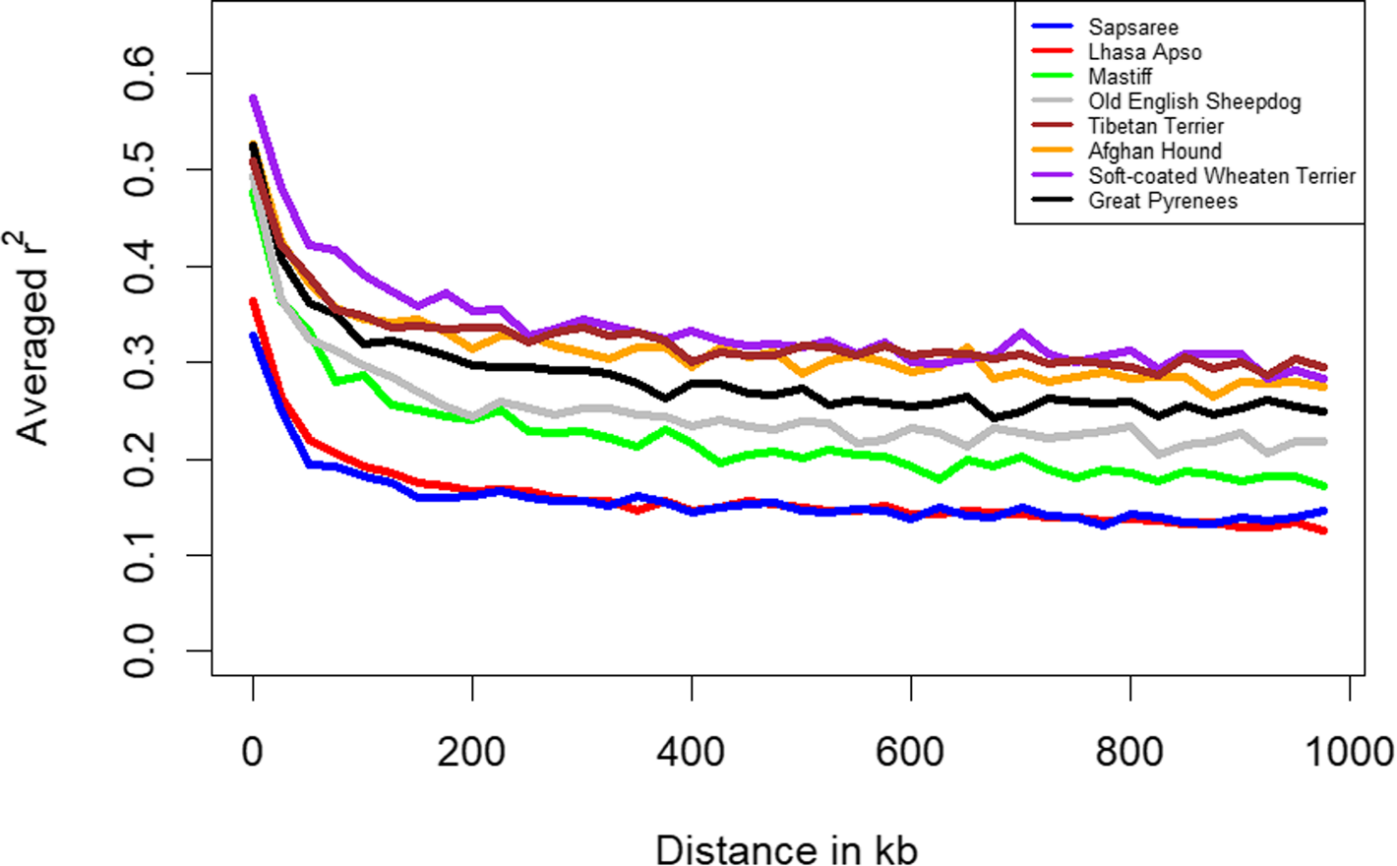
The decline in genome-wide linkage disequilibrium (LD), estimated as a function of genomic distance by calculating r^2^ values between all pairs of SNPs with inter-SNP distances of less than 1 Mb. Lines are colored based on breeds

As LD is expected to decay with recombination and increase the physical distance between markers [48], Fig. 1 shows different estimates of genome-wide LD for each of the eight populations, and declines in LD with increasing genomic distance across and within breeds. However, the rates of decay were different among breeds. Large differences were observed between Sapsaree, Lhasa Apso, and the other breeds. LD dropped off rapidly over a short distance in all breeds. Sapsaree and Lhasa Apso showed the lowest average LD across the genome. The breeds with the highest average LD were the Soft-coated Wheaten Terrier at the short marker distance but, the Tibetan Terrier at the long-distance marker. However, the LD values of Tibetan Terrier and Soft-coated Wheaten Terrier were not significantly different toward the long-distance.

**Fig. 2:**
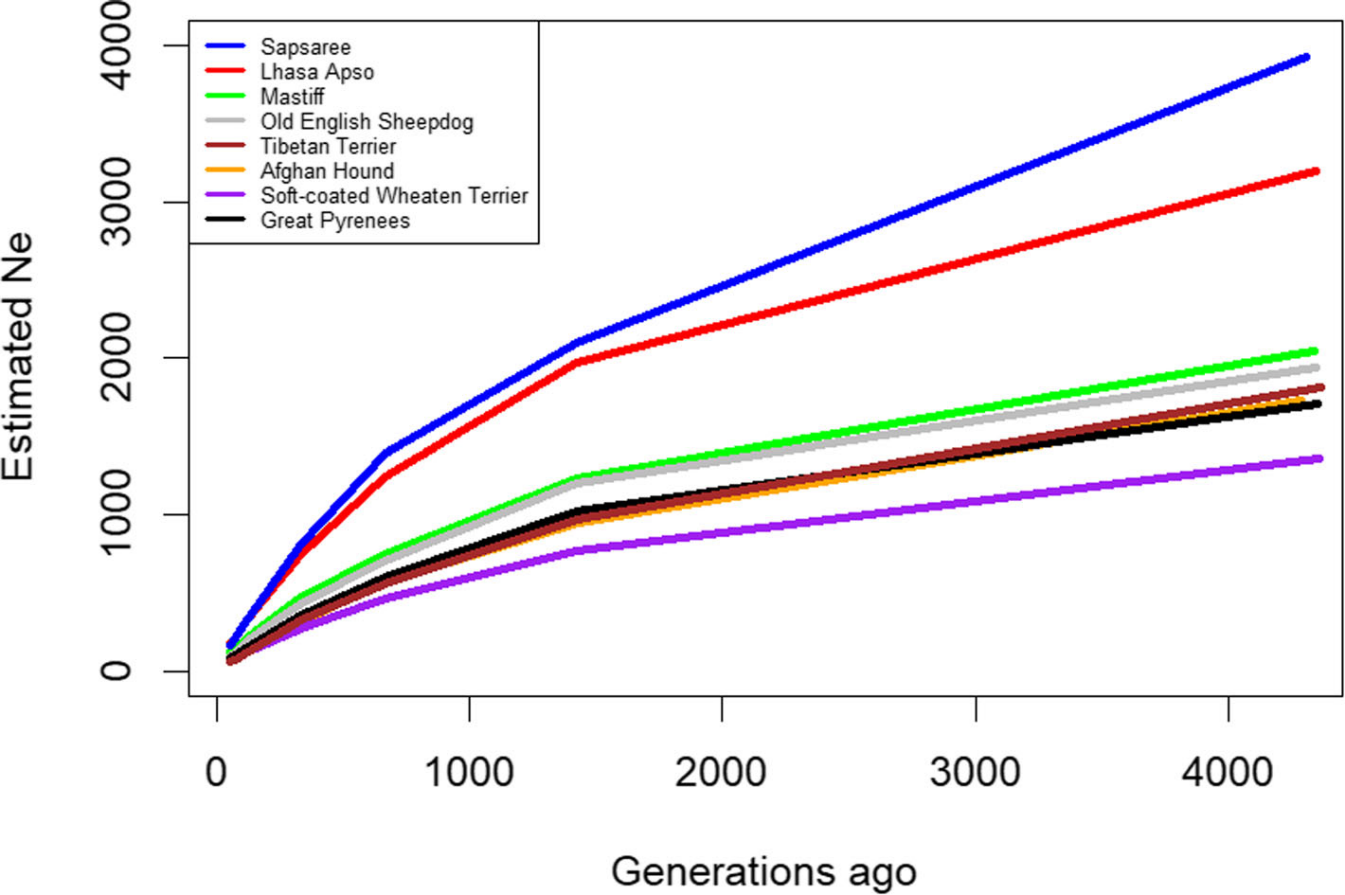
Trends in effective population size (N_e_) over generations based on LD (r^2^). Lines are colored based on breeds

The estimated effective population size (N_e_) at *t* generations ago is shown in Fig. 2. The results suggest that N_e_ was lower in the recent past compared with the ancient past (Fig. 2). Based on the genomic data 11 generations ago, the highest N_e_ was for Sapsaree which approximately 54 individuals, followed by Lhasa Apso (51 individuals) and the lowest N_e_ was approximately 17 individuals for the Tibetan Terrier (Fig. 2). In the more distant past of 1400 generations ago, the N_e_ was highest for Sapsaree approximately 2098 then 1966 for Lhasa Apso, and lowest for Soft-coated Wheaten terrier (approximately 764).

**Fig. 3:**
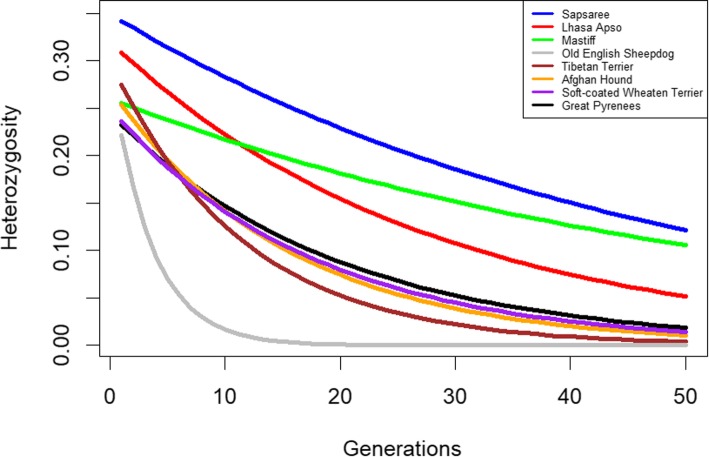
Estimated decay of heterozygosity over 50 generations. Lines are colored based on breeds

Heterozygosity was highest in the Sapsaree (0.342), followed by the Lhasa Apso (0.309) and Tibetan Terrier (0.273). The Old English Sheepdog (0.179) and Great Pyrenees (0.232) showed the lowest heterozygosity in the present generation (Fig. 3). Results suggest that heterozygosity will decline drastically in the future and is predicted to reduce by half within 25 generations. The estimated heterozygosity after 50 generations was also highest in the Sapsaree (0.118), with the Tibetan Terrier (0.003), Soft-coated Wheaten terrier (0.012), and Old English Sheepdog (0.000) showing the lowest values.

**Fig. 4:**
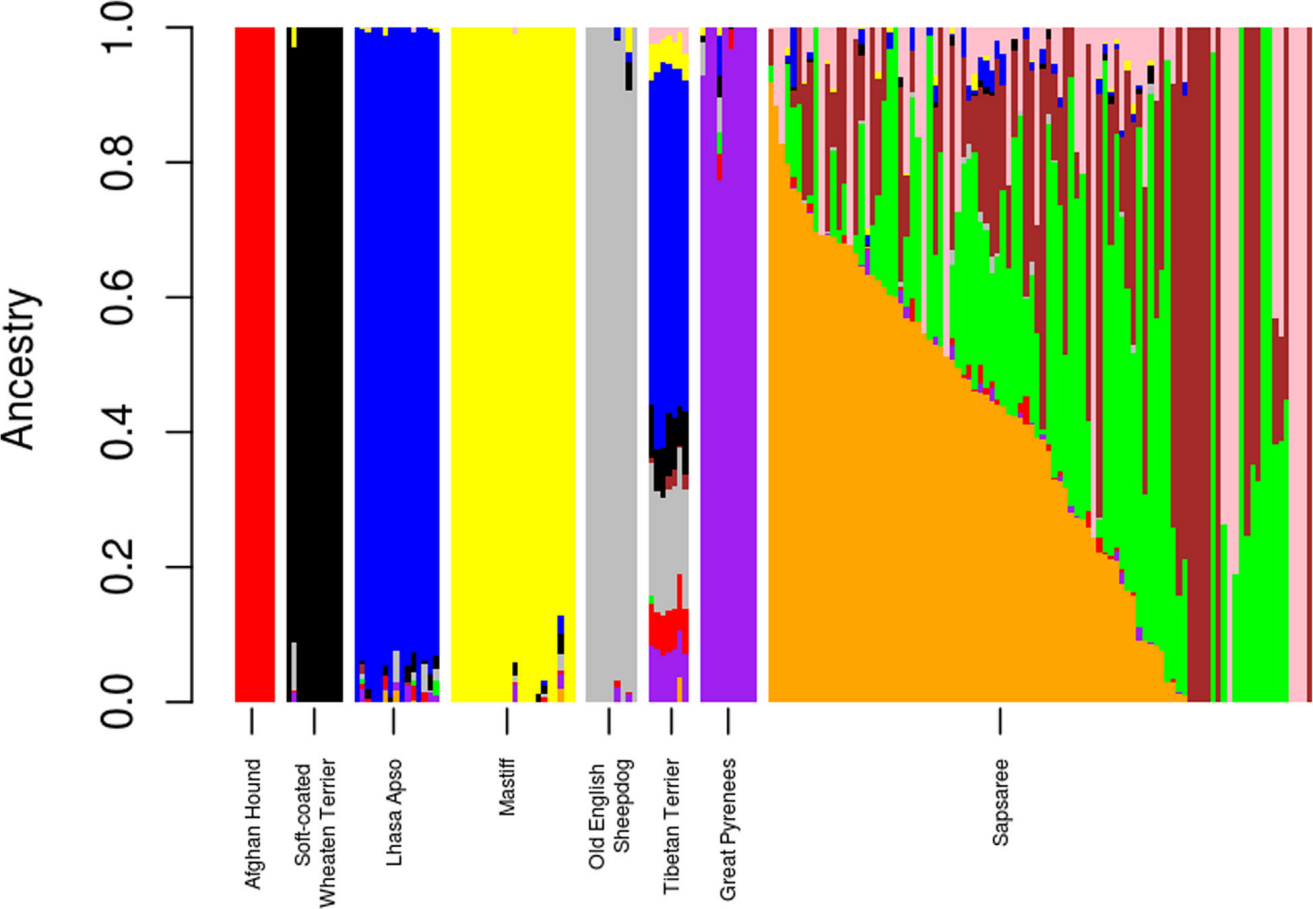
Population structure plots using K = 10 ancestry models. Each colored vertical line represents proportions of ancestral populations for each individual. K inferred the number of estimated ancestors and which differentiated by colors. Optimum K value was determined by Admixture’s cross-validation (CV) procedure. (Additional file 1: Figure S1)

Ancestry-based models of admixture analysis were used to show the genetic structure and admixture proportion of the canine ancestors (Fig. 4 and Additional file 3: Figure S3). Additional file 1: Figure S1 shows that the lowest CV error (0.583) was obtained at K = 10. The relationship of ancestry for Sapsaree and other breeds was visualized using K = 10, where K is the number of ancestors. Admixture models illustrated the greater degree of diversity and admixture in Sapsaree than the other breeds. Moreover, the admixture analysis was done with several other related dog breeds based on the genetic distance (Additional file 4: Figure S4) also revealed a greater genetic heterogeneity within the Sapsaree breed. Afghan Hound, Lhasa Apso, Great Pyrenees, Old English Sheepdog, Soft-coated Wheaten terrier, and Mastiff seem to have little or no admixture from other breeds, indicating that they have less remaining from other interacted ancestral breeds. Sapsaree indicated low levels of admixture with the Lhasa Apso and Tibetan terriers. Moreover, Sapsaree showed a small level of introgression with one of the oldest European breed Mastiffs ancestry, Great Pyrenees and the Old English Sheepdogs. However, admixture analysis indicated that major ancestries of Sapsaree were not shared with the other breeds used in this study.

**Fig. 5:**
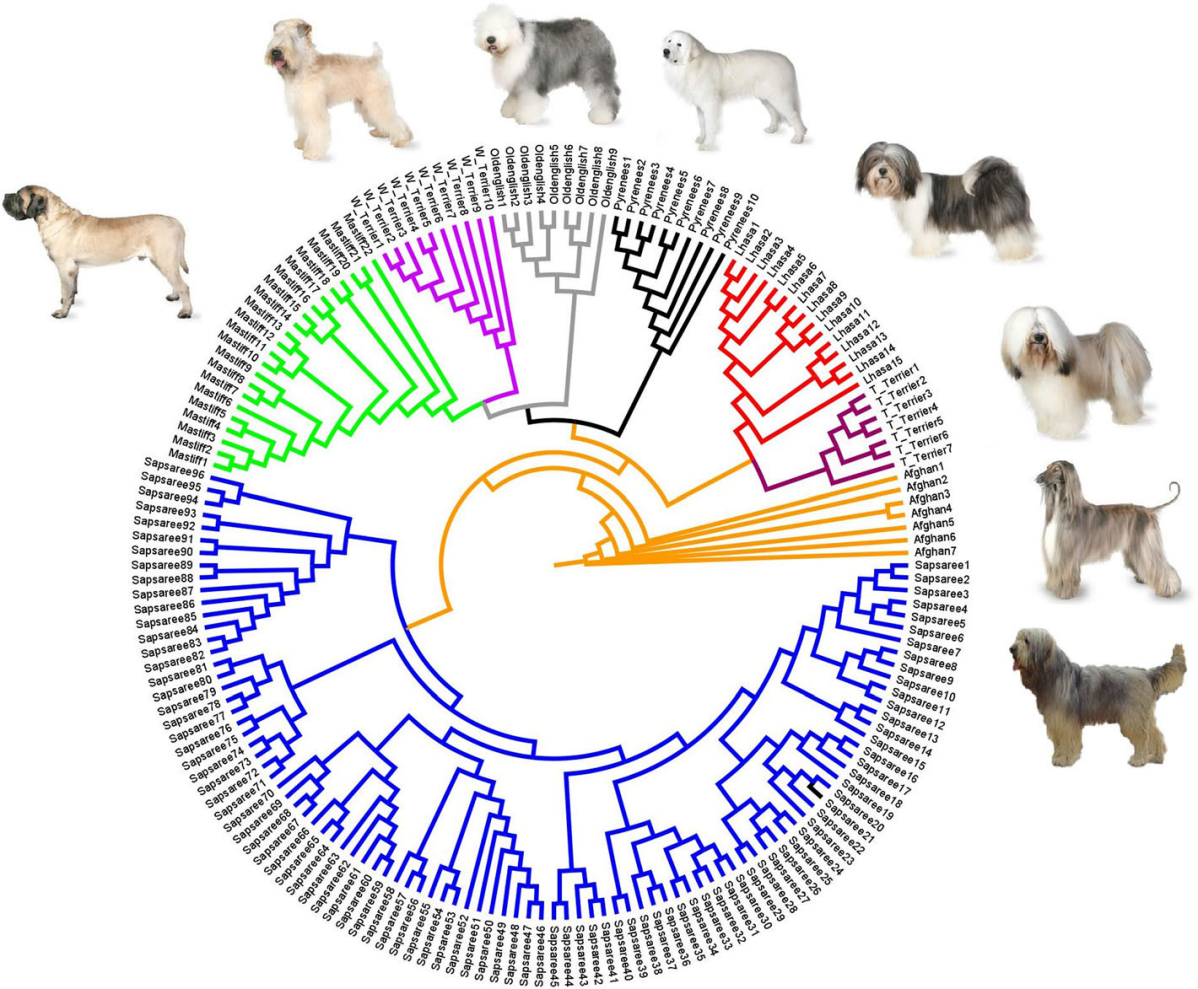
Phylogenetic tree of Sapsaree (blue) and other dog breeds (Afghan Hound, orange; Tibetan Terrier, magenta; Lhasa Apso, red; Great Pyrenees, black; Old English Sheepdog, gray; Soft-coated Wheaten terrier, purple; and Mastiff, green). The phylogenetic tree was rooted with the Afghan Hound. Canine images not drawn to scale. Afghan Hound, Tibetan Terrier, Lhasa Apso, Great Pyrenees, Old English Sheepdog, Soft-coated Wheaten terrier, and Mastiff images were obtained from http://dogtime.com/ and the Sapsaree image was obtained from http://www.worldlydogs.com/sapsaree.html

The phylogenetic tree clearly indicates a monophyletic clade of Sapsaree that is diverge from the other breeds, which supports the admixture analysis results (Fig. 5). The European breeds (Mastiff, Old English Sheepdog, Soft-coated Wheaten terrier, and Great Pyrenees) were grouped together in a single clade, and the Tibetan breeds (Tibetan Terrier and Lhasa Apso) comprise an adjacent monophyletic clade. The Afghan Hound was used as a root to construct the phylogenetic tree because it is an ancient breed, and more closer to a “real dog” than other domesticated breeds [7, 26, 49–51]. Our phylogenetic tree also indicates that the Afghan Hound is highly diverged from the other breeds.

**Fig. 6:**
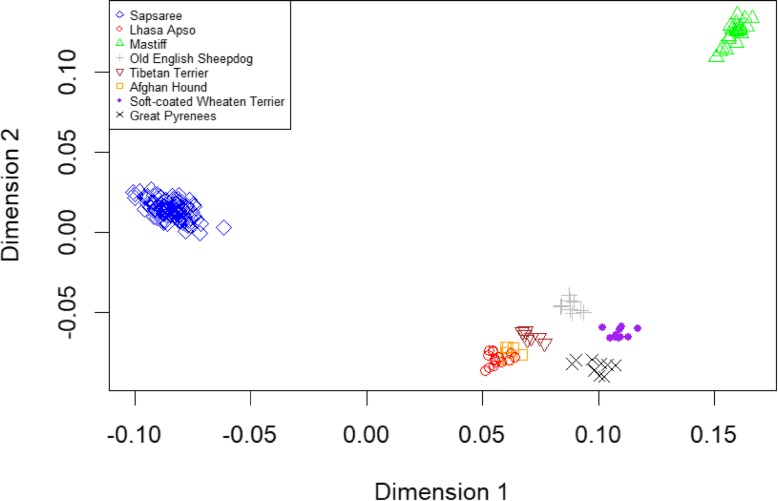
Clustering of breeds based on multidimensional scaling of genetic distance. Individuals are plotted on the first and second dimensions. Each dot represents an individual and colored shapes represent each dog breed

MDS analysis was used to visualize the quantitative estimates of genetic distance among the breeds (Fig. 6). Consistent with the admixture results, MDS also revealed that Sapsaree was clustered farthest from the other breeds, which supports assemblages into a single clade on the phylogenetic tree. However, Sapsaree clusters with the Mastiff, Old English Sheepdog, and Tibetan terrier when dimension 3 was plotted against dimension 4 (Additional file 2: Figure S2).

## Discussion

In this study, genome-wide SNP data was used to characterize the genetic diversity, population structure, and demographic history of an aboriginal Korean dog breed, the Sapsaree. The non-random association of genes at different loci is assessed as LD, which gives insight to the structure of present populations and evolutionary demographic events [52, 53]. Similar LD and N_e_ patterns in the Lhasa Apso and Sapsaree reflect their historical similarities [54]. Alam et al. [20] indicated that five generations ago, LD and N_e_ were approximately 0.2 and 64–75, respectively, which differs from our results. This variation may be due to discrepancies between samples and different algorithms used [6]. Ascertainment bias may have also caused the systematic deviation of population genetic structure from its theoretical expectations [55, 56]. N_e_ has long been recognized as a useful criterion for evaluating conservation status and threats to the genetic health of a population [57]. Meuwissen. [58] suggested that a threshold level of 50 or 100 for N_e_ would be necessary to maintain viable genetic diversity. Our results also emphasize that care should be taken to maintain the reasonable genetic diversity of the Sapsaree breed.

Ancient events, as well as the recent breeding program, can lead to dramatic changes in the genetic diversity among the individual dogs [6, 59–63]. Our analyses suggest that the Sapsaree has higher variance and discrete genetic compared to the other breeds studied here, consistent with the results of other studies [21, 23, 33]. Previous studies have also provided evidence that genetic diversity is high in dogs native to Korea [14, 21 9, 55] or East Asia [6, 64].

Heterozygosity is considered a useful parameter in estimating a population’s genetic diversity [32, 52, 65], and the Sapsaree has shown greater heterozygosity compared with foreign breeds [21, 23, 33]. One study indicated that the observed and expected mean heterozygosities in the Sapsaree were 0.460 and 0.543, respectively [23]. A recent study by Choi et al. [55] has suggested high heterozygosity (0.4) in Korean dogs (Poongsan, Donggyengi and Jindo). However, compared with the previous studies, there was low heterozygosity in the Sapsarees in this study. We were also determined that the Tibetan Terrier exhibits greater heterozygosity than the Mastiff [66] and alignment with the present results Mortlock et al. [67] showed multiple-locus heterozygosity of Mastiff was 0.206.

Population bottlenecks can dramatically reduce the genetic diversity of populations [68–71], and Sapsarees have experienced severe population bottlenecks during the Japanese colonial rule and the Korean War and subsequent economic crisis [18, 20, 23]. Interestingly, Sapsarees have still been able to maintain more genetic variation than other breeds.

Reductions in genetic variability or heterozygosity primarily depend on bottleneck size, rate of population growth, and mutation rate [72–74]. Although declines in genetic variability are expected following a bottleneck, variation may accumulate through mutations as the population size increases. Correspondingly, Kekkonen et al. [65] reported fairly high genetic diversity of white-tailed deer (*Odocoileus virginianus)* in Finland, even though the population was founded by four individuals in 1934 and remained isolated from other deer populations. In contrast, the German Leonberger breed had similar experience as which was nearly wiped out during World War I by violence and starvation. Their genetic variation drastically declined but was re-established in 1992 using five females and two males. However, their genetic variation was still low compared with other breeds [51, 69, 75].

Admixture, MDS, and phylogenetic analyses showed the unique diversity of the Sapsaree breed. Other studies have also found that native Korean dogs have substantially different genetic patterns than other foreign dog breeds [33, 55]. Furthermore, admixture analysis (Fig. 4 and, Additional file 3: Figure. S3 and Additional file 4:Figure S4) and structure analysis (Additional file 5: Figure S5) revealed a greater genetic heterogeneity within the Sapsaree compared to the other breeds. The consequences of the restoration process might be a reason for the increased genetic diversity of the breed. In 1986, the Sapsaree population was restored using eight individuals collected based on their similar characteristics with the original breed such as color and body shape. A system of non-restricted selection was then established to increase the population size [18, 76]. In alignment with the present results, several previous studies showed a greater genetic diversity of Sapsaree compared with foreign dog breeds [21, 23, 33]. Moreover, a small fraction of Sapsaree deviated from major genetic patterns, also possibly as a consequence of recent restoration processes. Founder animals were collected based on phenotypic characteristics, which might be lead some dogs having distance genetic pattern from majority of the Sapsaree population.

Correspondingly, Han et al. [22] showed that Sapsarees have greater genetic diversity based on several morphological traits such as tongue spots, dewclaws, tail-set, and coat, nose, and eye color. The Coat color of the Sapsaree also revealed the heterogeneous nature of the breed, indicating two distinct group of blue and yellow including several subdivisions such as blue black, grey black, deep yellow, yellow and light yellow [77]. On the other hand, some studies have also shown discrete phenotypic diversity such as Kim et al. [19] revealed that they can be divided into two groups based on gene expression patterns for physiological activities. Accordingly, the results suggest that systematic approach is needed to select the individuals for breeding to established the breed while ensuring the authenticity.

There was evidence of introgression into Sapsaree in the admixture analysis, which might have occurred prior to the restoration process when the population levels were low. Introgression from non-tested breeds could also have contributed to the high levels of genetic diversity noted in the Sapsaree. The admixture and MDS analyses provide compelling evidence that the ancestor of the Sapsaree is related to Tibetan long-haired breeds. The Tibetan Terrier and Lhasa Apso are native to Tibet, where they lived in nobles palaces and Buddhist monasteries as watch dogs, companions, and ‘good luck charms’. There are definitive evidences that which used as a special gift, tokens of esteem and good fortune when spreading the Buddhism [78–82]. Buddhism was introduced to Korea in fourth century CE [83, 84], and the introgression of Tibetan dog breeds might be an outcome of that relationship. Additionally, our results suggested the admixture of European dog breeds, which were introduced to the Korean peninsula as a result of cultural exchange. Christianity invaded Korea from Europe during the eighteenth century [81, 85], and some European dog breeds accompanied those missions. Afterwards, numerous European delegations and military correspondence with Korea occurred during World War I and the Korean War [86–88]. Furthermore, the Silk Road was a historical network of international trade routes from ancient China to Europe, stretching from Korea and Japan to the Mediterranean Sea. In addition to silk as the major commodity, companion animals were also exchanged on this route [89–91]. Comas et al. [92] suggested that genetic diversity was also traded along the Silk Road between Europe and eastern Asia. Consistent with our phylogenetic results, vonHoldt et al. [26] illustrated that European dog breeds, such as the Mastiff and Old English Sheepdog, are phylogenetically clustered, while Choi et al. [55] showed that the Tibetean Terrier and Lhasa Apso grouped into a single clade. Although, Jeong at al [23] suggested a great genetic distance between Sapsaree and the European breeds, their structure analysis showed a low level of genetic sharing among them, which support the current findings.

## Conclusions

Our results provide novel information regarding the genetic diversity and population structure of the native Korean dog, Sapsaree. Consistent with previous studies, our results also revealed higher genetic diversity in Sapsarees compared with other breeds. The majority of the breed showed a discrete genetic pattern, while a small fraction was genetically divergent and might be a consequence of recent restoration process. The N_e_ of the breed has declined drastically and is currently insufficient to maintain long-term viability of genetic diversity. Therefore, we suggest a strategic and systematic approach to ensure the purity and genetic diversity of the Sapsaree breed, a Korean natural treasure. Admixture analysis revealed a complex pattern of Sapsaree, where major ancestries were not shared with the other breeds analyzed in this study. LD, N_e_, genetic diversity, and population structural analyses indicate a relationship between Sapsaree and the long-haired breeds Tibetan Terrier and Lhasa Apso. Introgression from European breeds was also revealed.

## Methods

### Animals, genotyping, and quality control

All research methods were approved by the Institutional Animal Care and Use Committee of the Rural Development Administration in South Korea. To investigate the genetic origin of the Sapsaree breed, we selected seven foreign dog breeds analyzed in a previous study Shannon et al. [34] based on their phenotypes, such as long haired and body conformation [35]. The Sapsaree (*n* = 96), Lhasa Apso (*n* = 15), Great Pyrenees (*n* = 10), Tibetan Terrier (*n* = 7), Afghan Hound (*n* = 7), Old English Sheepdog (*n* = 9), Soft-coated Wheaten Terrier (n = 10), and Mastiff (*n* = 22) dog breeds were categorized as ancient or modern breeds according to Vonholdt et al. [26] and Parker et al. [13]. Based on a memorandum of understanding between the research team and the Sapsaree conservation center, blood samples were collected by veterinarians in an ethical manner according to the animal health and welfare guidelines (Approval numbers: 2016–177).

Samples were genotyped using Illumina CanineSNP20 BeadChip. Other breeds were genotyped by [34] using the Illumina CanineHD array and merged into our dataset. The CanineSNP20 BeadChip is Illumina’s first non-human standard genotyping panel contains more than 22,000 evenly spaced and validated SNP probes derived from the CanFam2.0 assembly. The CanineHD Genotyping BeadChip contains more than 170,000 markers placed also on the CanFam2.0 reference sequence. This presents an average of greater than 70 markers per megabase (Mb), providing ample SNP density for robust within-breed association and copy number variation (CNV) studies (www.illumina.com). The quality of SNP data was maintained with the use of PLINK 1.9 [36] to filter SNPs with low call rates (< 90%) or missing genotypes (> 10%). To reduce bias, the number of minor allele frequencies was limited to 1%, and deviations from Hardy-Weinberg equilibrium (*P* > 0.001) were also excluded [37]. Non-autosomal SNPs were also removed from analyses.

### Linkage disequilibrium, effective population size, and heterozygosity

The extent of LD between markers was measured using the squared correlation coefficient of allele frequencies at pairs of loci (r^2^) with inter-SNP distance within 1 Mb, both within a given breed and across all breeds [38]. Pairwise LD between adjacent SNPs was calculated for each chromosome using the default PLINK V1.9 approach [39]. Effective population size (N_e_) was estimated based on the LD value (r^2^) using the SNeP V1.1 tool [29, 40–42]. Heterozygosity over the next 50 generations was estimated as described by [43]. Statistical software package R [44] was used to produce graphical representations. Wright–Fisher model was used to calculate the forward derivation of heterozygosity, assuming that N diploid parents produce a large number of gametes, these gametes randomly unite to produce a large number of zygotes, and from these zygotes, N progeny are randomly chosen to form the next generation [43].

### Genetic diversity and population structure

Population structure and genetic diversity were studied using multi-dimensional scaling (MDS) analysis, ancestor’s admixture prediction, and phylogenetic comparisons. To create a matrix representation of interbreed relationships, MDS algorithms of pairwise genetic distances were implemented in PLINK [39] and depicted as coordinates in R. Population substructures and the extent of mixture between ancestral populations of Sapsaree and unrelated individuals of other studied breeds were evaluated through the model-based clustering algorithm using ADMIXTURE v.1.23 [45]. To reduce prediction error, admixture’s cross-validation (CV) procedure was used to determine the optimal K-value by minimizing CV error. These results were graphed using R. A phylogenetic tree was developed using the SNPhylo software package and illustrated using FigTree software v. 1.4.2 to infer the evolutionary relationships among breeds [46, 47].

## Additional files

Additional file 1: **Figure S1.** Cross-validation plot of admixture analysis. The *x*-axis represents the number of clusters (K) in the model and the *y*-axis represents cross-validation error values. (DOCX 33 kb)

Additional file 2: **Figure S2.** Clustering of animals from Sapsaree and other selected breeds based on multidimensional scaling of genetic distance. Individuals are plotted for the third and fourth dimension. (DOCX 22 kb)

Additional file 3: **Figure S3.** Population structure plots using K = 8 and K = 11 ancestry models. Each colored vertical line represents proportions of ancestral populations for each individual. K inferred the number of estimated ancestors and which differentiated by colors. (DOCX 214 kb)

Additional file 4: **Figure S4.** Ancestry model for Sapsaree including related dog breeds based on the genetic distance. Each colored vertical line represents proportions of ancestral populations for each individual. K inferred the number of estimated ancestors and which differentiated by colors. Optimum K value (K = 16) was determined by Admixture’s cross-validation (CV) procedure. (DOCX 64 kb)

Additional file 5: **Figure S5.** The population structure bar plots generated by STRUCTURE software at K = 8. (DOCX 73 kb)

Additional file 6: **Figure S6.** Heat map of relatedness between the individuals of Sapsaree and other studied breeds. (DOCX 95 kb)

Additional file 7: **File S7.** Genotype information of Sapsaree. (BED 524 kb)

Additional file 8: **File S8.** Genotype information of Sapsaree. (BIM 697 kb)

Additional file 9: **File S9.** Genotype information of Sapsaree. (FAM 2 kb)

## Data Availability

All data generated or and analyzed during this study are included in this published article [and its supplementary information files]. Sapsaree genotype data have been uploaded as Additional files 6, 7, 8 and 9. Genotype data of the other breeds are available from Shannon et al., 2015 (10.5061/dryad.v9t5h).

## Abbreviations

Afghan: Afghan Hound
CV: Cross-validation
H: Heterozygosity
LD: Linkage Disequilibrium
Lhasa: Lhasa Apso
MDS: Multi-dimensional scaling
N_e_: Effective Population size
Oldenglish: Old English Sheepdog
Pyrenees: Great Pyrenees
T_Terrier: Tibetan Terrier
W_Terrier: Soft-coated Wheaten Terrier
