# Supplementary figures and images for: Genetic diversity and population structure of the Sapsaree, a native Korean dog breed
Source: BMC Genet. 2019 Aug 5;20:66. doi: 10.1186/s12863-019-0757-5 (PMC6683530; doi:10.1186/s12863-019-0757-5)

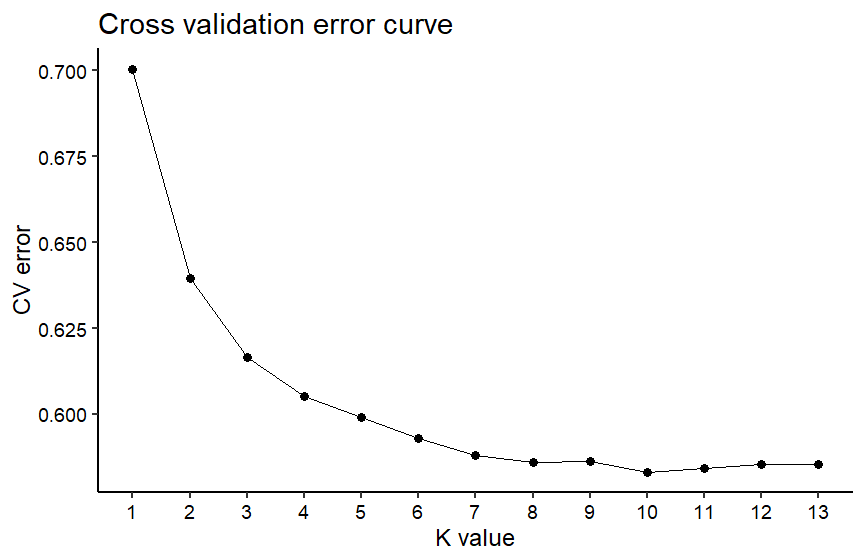
**Additional file 1: Figure S1.**

Supplement: Supplementary file 1 — Figure S1. Cross-validation plot of admixture analysis. The x-axis represents the number of clusters (K) in the model and the y-axis represents cross-validation error values. (DOCX 33 kb) [file 12863_2019_757_MOESM1_ESM.docx]

**Additional file 2: Figure S2**


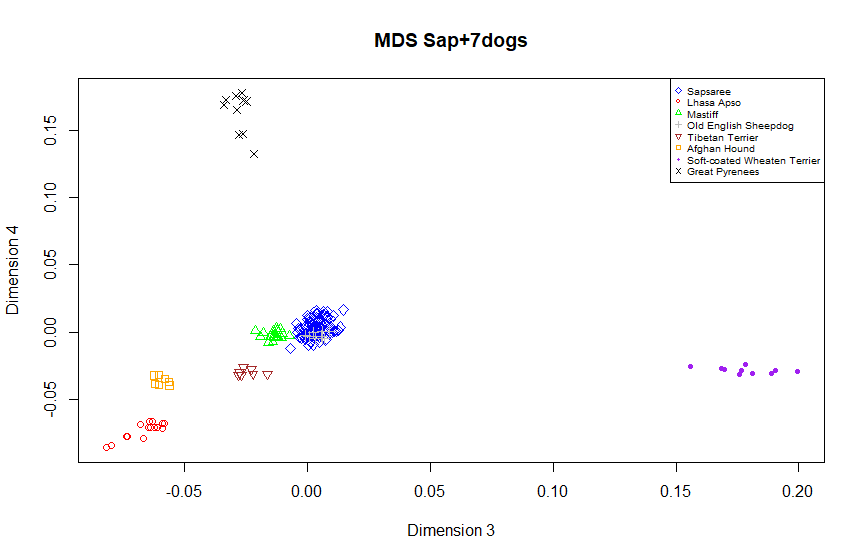

Supplement: Supplementary file 2 — Figure S2. Clustering of animals from Sapsaree and other selected breeds based on multidimensional scaling of genetic distance. Individuals are plotted for the third and fourth dimension. (DOCX 22 kb) [file 12863_2019_757_MOESM2_ESM.docx]

**Additional file 3: Figure S3.**


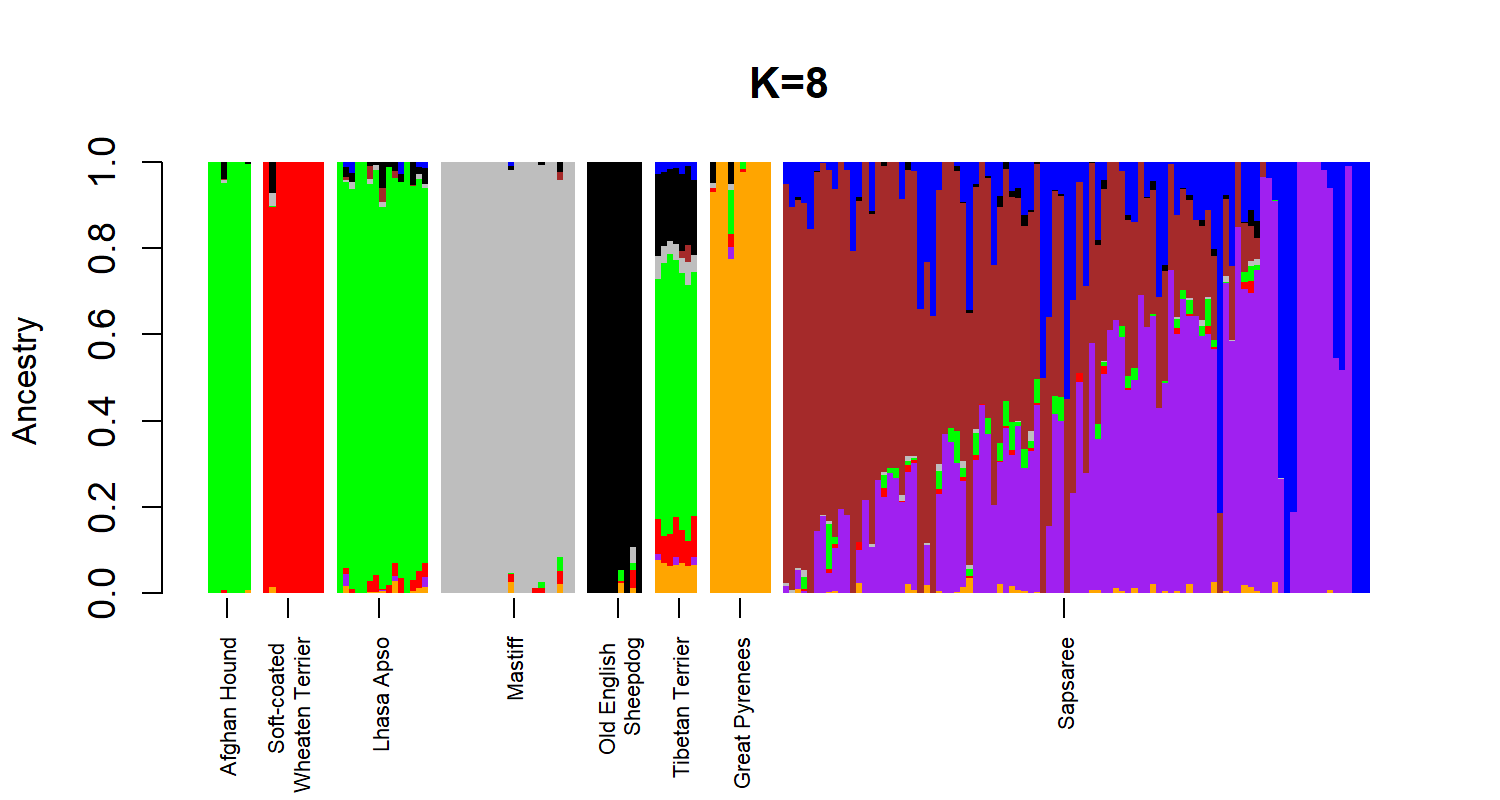

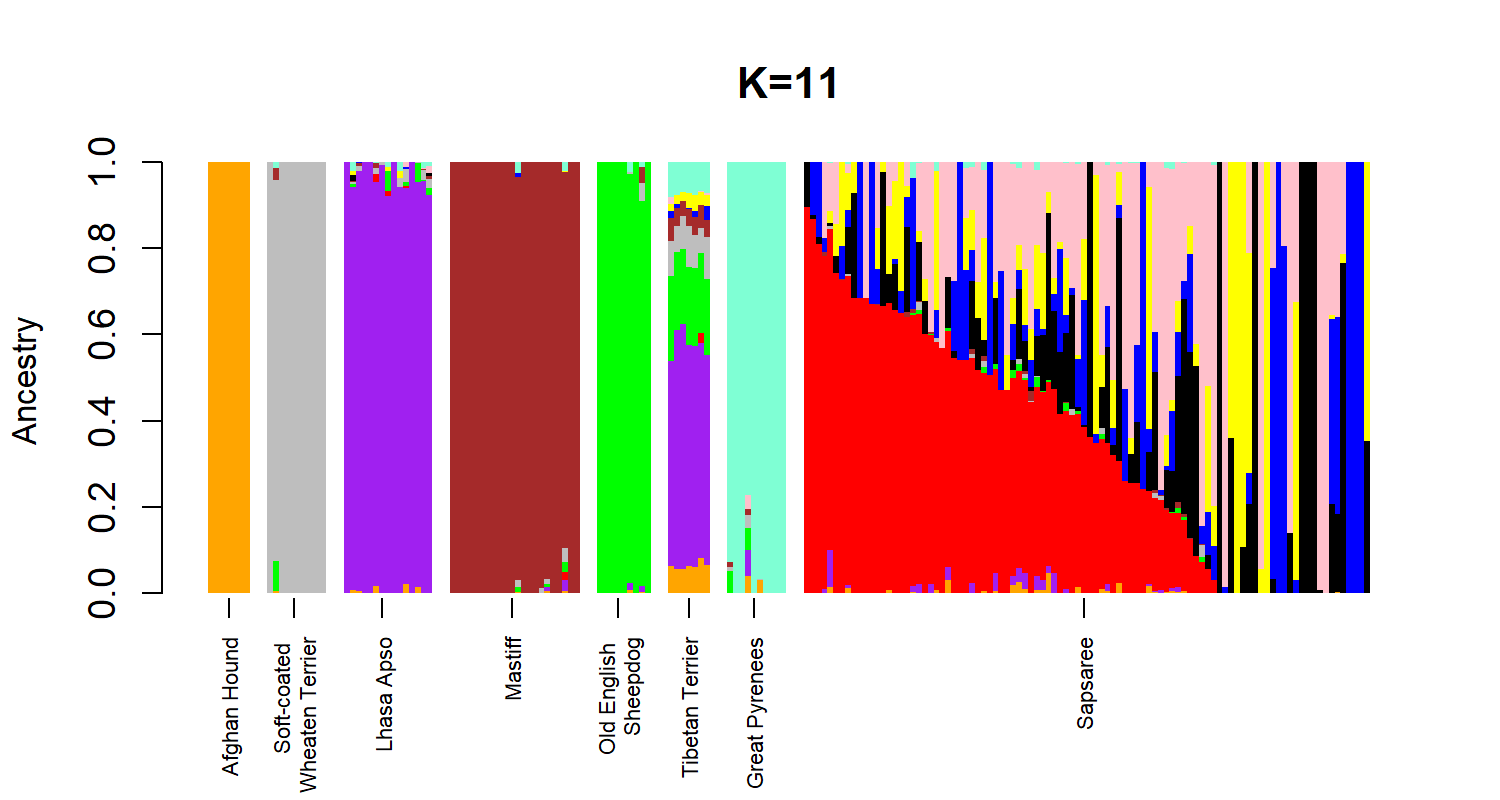

Supplement: Supplementary file 3 — Figure S3. Population structure plots using K = 8 and K = 11 ancestry models. Each colored vertical line represents proportions of ancestral populations for each individual. K inferred the number of estimated ancestors and which differentiated by colors. (DOCX 214 kb) [file 12863_2019_757_MOESM3_ESM.docx]

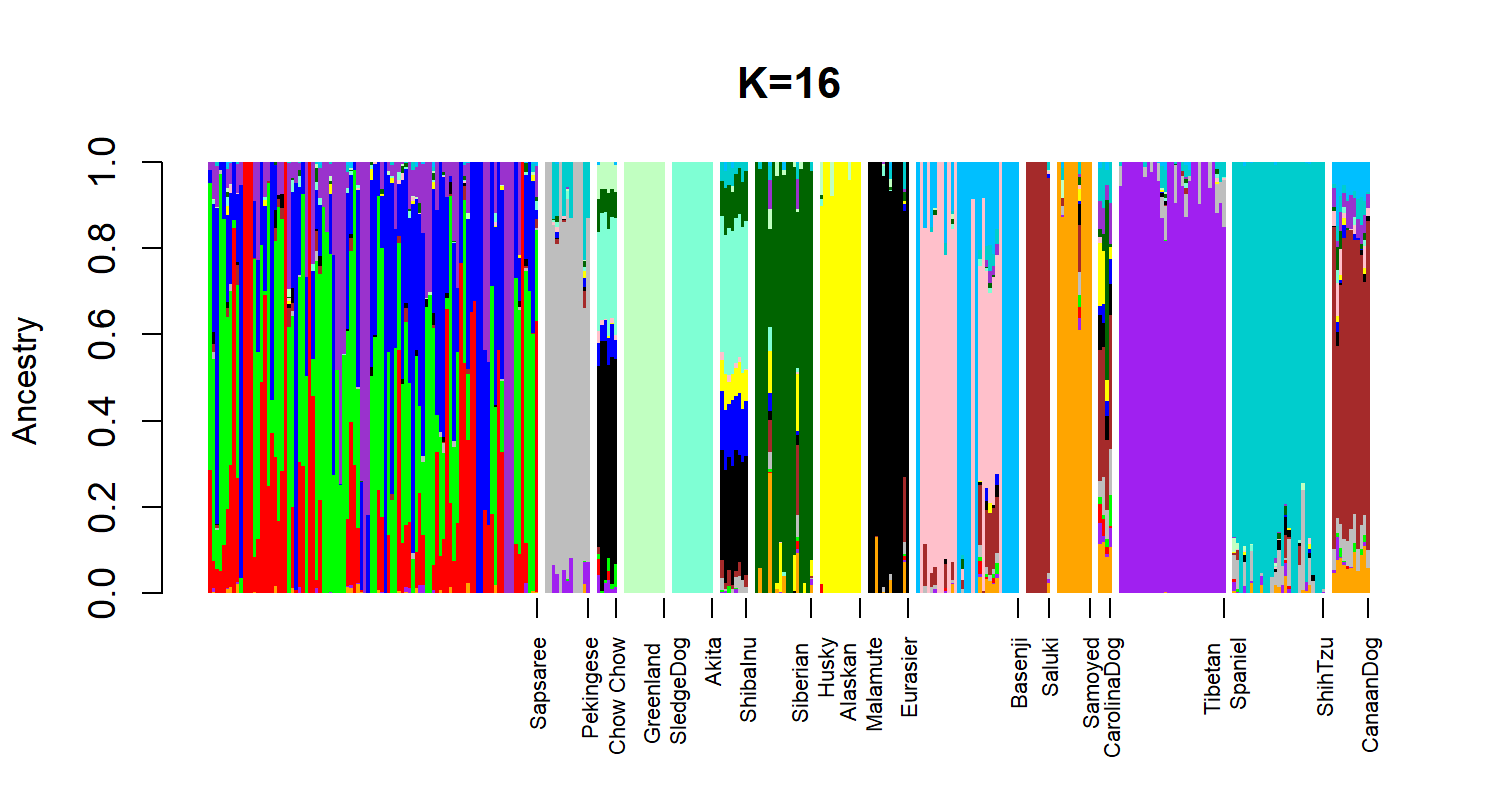
**Additional file 4: Figure S4.**

Supplement: Supplementary file 4 — Figure S4. Ancestry model for Sapsaree including related dog breeds based on the genetic distance. Each colored vertical line represents proportions of ancestral populations for each individual. K inferred the number of estimated ancestors and which differentiated by colors. Optimum K value (K = 16) was determined by Admixture’s cross-validation (CV) procedure. (DOCX 64 kb) [file 12863_2019_757_MOESM4_ESM.docx]

**Additional file 5: figure S5.**


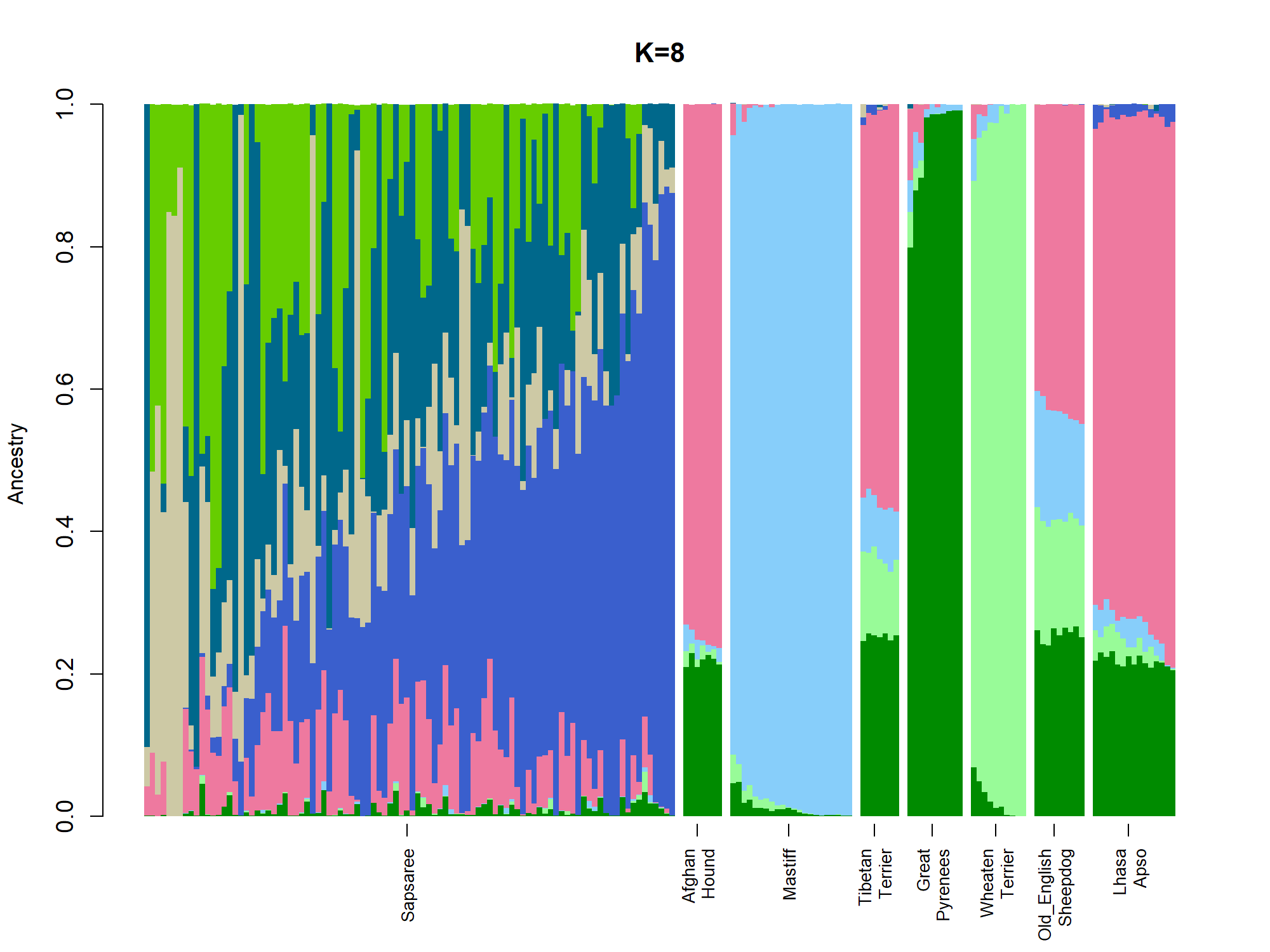

Supplement: Supplementary file 5 — Figure S5. The population structure bar plots generated by STRUCTURE software at K = 8. (DOCX 73 kb) [file 12863_2019_757_MOESM5_ESM.docx]

**Additional file 6: figure S6.**


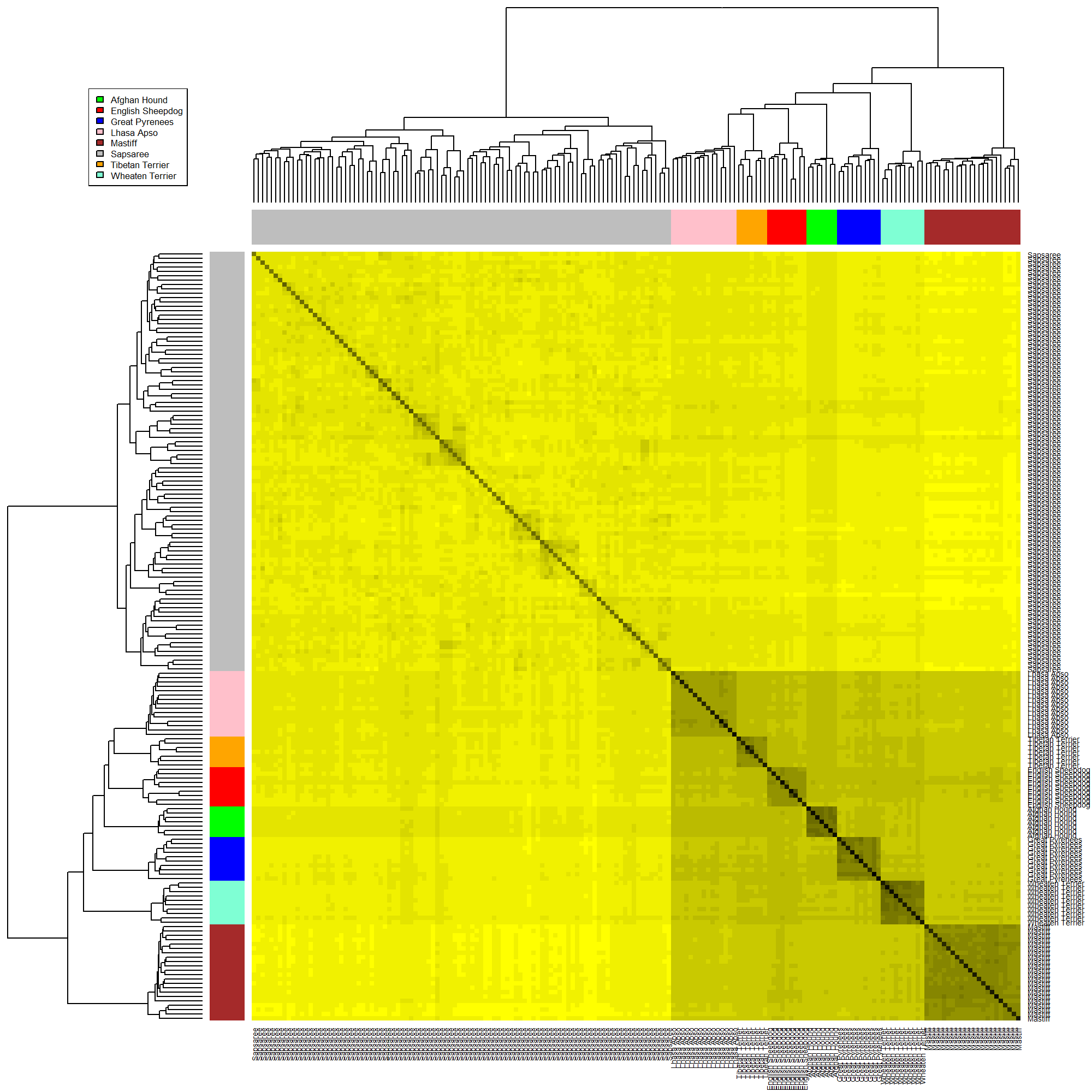

Supplement: Supplementary file 6 — Figure S6. Heat map of relatedness between the individuals of Sapsaree and other studied breeds. (DOCX 95 kb) [file 12863_2019_757_MOESM6_ESM.docx]
